# Electro-Conductive Membranes for Permeation Enhancement and Fouling Mitigation: A Short Review

**DOI:** 10.3390/membranes7030039

**Published:** 2017-07-28

**Authors:** Patrizia Formoso, Elvira Pantuso, Giovanni De Filpo, Fiore Pasquale Nicoletta

**Affiliations:** 1 Department of Pharmacy, Health and Nutritional Sciences, University of Calabria, I-87036 Rende (CS), Italy; patrizia.formoso@unical.it (P.F.); elvirapnt.ep@gmail.com (E.P.); fiore.nicoletta@unical.it (F.P.N.); 2 Department of Chemistry and Chemical Technologies, University of Calabria, I-87036 Rende (CS), Italy

**Keywords:** membrane fouling, membrane cleaning, stimuli responsive polymer membranes, electro-responsive membranes, fouling mitigation, permeation enhancement

## Abstract

The research on electro-conductive membranes has expanded in recent years. These membranes have strong prospective as key components in next generation water treatment plants because they are engineered in order to enhance their performance in terms of separation, flux, fouling potential, and permselectivity. The present review summarizes recent developments in the preparation of electro-conductive membranes and the mechanisms of their response to external electric voltages in order to obtain an improvement in permeation and mitigation in the fouling growth. In particular, this paper deals with the properties of electro-conductive polymers and the preparation of electro-conductive polymer membranes with a focus on responsive membranes based on polyaniline, polypyrrole and carbon nanotubes. Then, some examples of electro-conductive membranes for permeation enhancement and fouling mitigation by electrostatic repulsion, hydrogen peroxide generation and electrochemical oxidation will be presented.

## 1. Introduction

The most important property for assessing the quality of a separation process through a membrane is its selectivity for a compound on another compound, also known as permselectivity.

Higher permeability values require lower membrane areas to separate a given compound, and a very good selectivity leads to higher purity products and, accordingly, to optimized values of rejection [1,2]. Nevertheless, the onset of fouling, due to the deposition/adsorption of particulate and soluble materials on membrane surfaces with time, causes a decrease in the permeability and selectivity with detrimental effects on membrane processes.

The present review summarizes recent developments in the preparation of electro-conductive membranes and the mechanisms of their response to external electric voltages in order to obtain an improvement in permeation and mitigation in the fouling growth. In particular, this paper deals with the properties of electro-conductive polymers and the preparation of electro-conductive polymer membranes with a focus to responsive membranes based on polyaniline, polypyrrole and carbon nanotubes, which represent the most used electro-conductive polymers. Then, some examples of electro-conductive membranes for permeation enhancement and mitigation of membrane fouling by electrostatic repulsion, hydrogen peroxide generation and electrochemical oxidation will be presented.

## 2. Electro-Conductive Membranes

Membranes are selective barriers able to separate components with different sizes or physical/chemical properties. The efficiency of a membrane separation process depends on the selectivity and permeability of used membranes. Selectivity, i.e., the ability to separate solutes, contaminants and particles with different sizes or physical/chemical properties is determined by the rejection of the unwanted compound and the permeation of desired compound. Generally, the membrane selectivity depends on the affinity between the substances and membrane porous surface, effective pore size and distribution. The permeability of a membrane is typically quantified by the trans-membrane flux and influenced by the pore size and surface properties of membranes [3]. Therefore, the performance of porous membranes can be weakened upon adsorption and deposition of foulants, present in the feed mixtures, on the porous surface.

Thus, for various size-based membrane separation applications, tunable/switchable pore sizes are required to achieve adjustable selectivity and permeability in response to external stimuli (single or multiple) or to environmental changes in feed conditions.

All responsive membranes have channels able to self-regulate their permselectivity in response to environmental stimuli, such as temperature, pH, specific molecules/ions, light, electric/magnetic fields, ionic strength, and redox reactions [4,5,6,7,8,9,10,11,12,13,14,15,16,17,18]. A common method to add responsiveness to a membrane is the use of stimuli-responsive polymers, copolymers and mixtures of polymers and additives during or after the membrane formation [19].

Such responsive membranes can act as smart valves, allowing an on demand flux control by dynamic modification of their structure and transport properties (e.g., permselectivity and hydrophilicity). In such a way, it is possible to enable a fouling mitigation and tunable self-cleaning membrane surfaces without the use of physical/chemical cleaning methods required for membranes under normal operating conditions.

Electro-Responsive Polymer Membranes (ERPMs), i.e., polymer membranes able to respond to an electric potential, can be obtained by membrane functionalization with custom-designed electrically conductive polymers.

According such a rationale several biosensors, electronic devices, and biomimetic devices have been prepared from electrically conductive polymer membranes [20,21,22,23]. The specific reactivity/polarity/conformation of used conducting polymers, virgin or properly functionalized for a better integration in the pore structure, enables an electro-responsiveness in filtration membranes usually adopted for water treatment [24,25].

Porous membranes used for microfiltration (MF) and ultrafiltration (UF) processes are characterized by a pore size ranging from 0.1 μm to 10 μm and 2 nm to 100 nm, respectively. The coating with a thin and selective polymer layer enables their use in nanofiltration (NF), reverse osmosis (RO), desalinization [26], and crystallization [27] processes. Some of such membranes can gain a responsive behavior by grafting electro-responsive polymers onto their surface or inside pore walls [3,28].

Unfortunately, only a limited number of electrically conductive polymers are suitable to be easily integrated into the most common production process of composite membranes, such as Non-Solvent Induced Phase Separation for MF and UF membranes, and interfacial polymerization or coating for NF and RO membranes. Recently, a different method based on using responsive polymer self-assembly has been proposed for functionalizing commercial membranes (either by a post-processing procedure or in a single step process) and improving permselectivity and fouling potential [29,30].

### 2.1. Electro-Conductive Polymers

In water treatment applications, it is important that membranes show opportune surface structure (e.g., pore size and distribution), purposeful hydrophilicity, adequate chemical-physical properties, high mechanical stability, and long term durability. ERPMs can contain specific organic and/or inorganic solid nanofillers in their porous structure (nanocomposite or hybrid membranes) to give or enhance the membrane properties that would otherwise not be met by the conducting polymer alone [31].

Electro-conductive polymers can be classified according to the movement of electric charges [32] in:

1. Intrinsic electro-conductive polymers, characterized by conjugated π-π or p-π systems;
2. Redox polymers that possess redox potentials within their structure groups (reduction/oxidation capacity).

The electronic transport in intrinsic electro-conductive polymers is due to the electron transfer from π type bonds to nearby simple σ bonds, due to the repulsion effect of same type charges. In presence of heteroatoms (N, S or O type) within the macromolecular polymer chains, the electron transfer is from π type bonds to non-participating p electrons of the heteroatoms that, moving to σ single bonds, further induces the movement of π electrons from the nearby double bonds by electrostatic repulsion effect. The conductivity of intrinsic conductive polymers significantly increases by oxidative and reductive doping (p-doping and n-doping, respectively).

The electronic movement in the case of redox polymers is gained through “donor-acceptor” reversible chemical reactions, according to Equation (1):

(1) $$Ox+n\;e^{-}\;⇄\;Red$$

if the chemical groups, with their redox potential distributed within the macromolecular structure, enable electronic jumps between groups [32].

Table 1 shows the most representative electro-conductive polymers, but not their numerous derivatives. Nevertheless, there are few stable conducting polymers in harsh aqueous environment and most of recent studies on ERPM are limited to the use of commercially available conducting polymers.

### 2.2. Preparation of Electro-Responsive Polymer Membranes

Surface material research has recently led to the manufacturing of many smart membranes by either chemical bonds or physical incorporation of electro-responsive materials on porous membrane substrates. It is well known that in typical processes for preparation of conventional membranes, the enrichment of membranes with conductive polymers is limited by the doping amounts used to improve membrane electro-responsiveness without loss of the mechanical properties. Electro-responsive membranes can be obtained from casting of conductive polymer thin films or self-assembling of monolayers onto the membrane surface by different methods such as plasma deposition, chemical vapour deposition, spin coating, chemical and electrochemical reactions, and layer-by-layer assembly [3]. The physical coating with a conductive polymer generally leads to variations in membrane swelling degree and changes in membrane permselectivity [33,34]. Alternatively, functional polymers, as well known as polymer brushes**,** can be attached in a controlled manner on the membrane surfaces or within membrane pores by physical adsorption or covalent bonds [35,36,37,38]. Polymer brushes can be covalently attached to membrane surfaces and pores either by ‘grafting-from’ methods or by ‘grafting-to’ techniques. In the ‘grafting-from’ methods, functional monomers are polymerized onto active sites present on the membrane pores and surface. The ‘grafting-from’ method is considered very advantageous because the presence of linear polymers or crosslinked networks in the pores can reduce the steric hindrance of neighbouring bonded polymer chains [39,40]. On the contrary, in the ‘grafting-to’ methods responsive membranes are fabricated by chemical/physical incorporation of opportunely end-functionalized polymers that can react onto the desired surfaces. In both grafting methods, the presence of an electro-responsiveness in the grafted polymer brushes can be used to alter the chain conformation and lead to responsive surfaces with an electro-tuneable permselectivity. Grafting can be usually induced by plasma, photo-irradiation, redox reactions, temperature and controlled radical reactions such as reversible addition fragmentation chain transfer polymerization and atom transfer radical polymerization. Polymer self-assembly methods to prepare ERPM membrane in a single step are limited by the difficulties generally found in the synthesis of conductive copolymers. Recently, polymer self-assembly methods have used amphiphilic copolymers in order to prevent membrane fouling and retain permeability [41,42,43,44,45]. Barghi et al. [46] synthesized a flexible, biocompatible, semi-hydrophilic, and electro-conductive membrane by crosslinking copolymerization of a highly electro-conductive monomer (hydroxymethyl-3,4-ethylenedioxy thiophene, HMEDOT) with a highly mechanical resistant polyamide (polytetramethylene-N-hydroxyethyl adipamine, PTMHEA) opportunely hydrophilized with acetaldehyde and in situ polymerized by an oxidative plasma treatment. The PHMEDOT homopolymer grafted onto the PHMEDOT-*co*-PTMHEA surface reduced considerably the copolymer electrical resistance both in dry and wet conditions (105 kΩ cm^−2^ and 2 kΩ cm^−2^, respectively). Pore size and distribution, roughness, and water flux were finely controlled by changing the thickness of PHMEDOT homopolymer.

#### 2.2.1. Responsive Membranes Based on Polyaniline

Polyaniline (PANI) is one of the most investigated conductive polymers because of its long term environmental stability, high conductivity, and relative low cost. The high chemical selectivity of PANI and its composites makes them particularly attractive as sensors for the detection of a number of gases and vapours, including methanol, ammonia, HCl, CHCl_3_, NO_2_, and CO [47,48]. Distinctive drawbacks, such as low solubility in the majority of solvents commonly used for membranes preparation, low mechanical flexibility, and thermal instability at temperatures above 160 °C, do not allow obtaining pure PANI membranes. Therefore, PANI-based responsive membranes are blends of PANI with other polymers suitable for membrane preparation. Polysulphone (PSF), polystyrene (PS), polypropylene (PP), cellulose (CEL) and its derivatives are some chemically inert polymers used for PANI-based membrane preparation. PANI-based membranes are mainly used in selective separation processes of gases and some chemical species from complex liquid solutions, in antistatic textile materials, biosensors, anticorrosive films, and electric and electronic devices (e.g., light emitting diodes and photovoltaic cells).

PSF/PANI-based membranes are designed for advanced separation of polar compounds from various mixtures and obtained from simultaneous formation of a PSF-based membrane and aniline polymerization within membranes in oxidative conditions. PANI results generally well distributed in the whole microporous structure and not only on membrane surface [49]. In addition, PS/PANI-based membranes can be obtained via phase inversion processes, whereas phase changes take place through precipitation in vapour phase [50]. Obviously, conductive properties of PS/PANI-based membranes depend on PS/PANI weight ratio within the composites. PP/PANI-based membranes maintain the microporous structure of the supporting polypropylene. Different pore diameters can be obtained as long as PANI is formed within the PP pores through soaking of supporting polymer films in aniline, followed by aniline oxidative polymerization using ammonium peroxydisulphate and HCl [51]. PP/PANI-based composites can be used for selective separation of chemical species from various liquid media through reverse osmosis, microfiltration, ultrafiltration, and nanofiltration processes. CEL/PANI nanocomposites can be prepared by in situ chemical oxidative polymerization routes of aniline within the fibre microstructure of CEL [52]. An enhancement in the PANI content inside membrane nanocomposites and a consequent increase in their electric conductivity is observed by increasing the reaction time. Longer reaction times give rise to PANI aggregation and formation of discontinuities within the nanocomposite structure with a consequent decrease of electric conductivity. Other CEL/PANI-based membranes can be prepared by deposition of a thin layer of PANI onto membrane interface (cellulose or its esters) by in situ oxidative polymerization of aniline [53]. Therefore, the electric conductivities of cellulose acetate/PANI membranes increase from 10^−3^ to 11 S m^−1^ and 98 S m^−1^ using liquid [54] and vapour [55] phase polymerization, respectively.

#### 2.2.2. Responsive Membranes Based on Polypyrrole

Polypyrrole (PPy) is characterized by very low conductivity and a low processability due to its poor mechanical strength. An appropriated doping with anions such as dodecylsulphate, chloride, sulphate and perchlorate can easily increase conductivity. Doped PPy shows a good chemical and thermal stability, a higher conductivity compared with other conductive polymers, and improved plasticity and elasticity by inclusion within polymer structure. PPy-based composite membranes are frequently used in concentration- (gas separation from complex mixtures and pervaporation) and electric potential-gradient processes (electro-dialysis). PPy is polymerized by electrochemical and chemical oxidative polymerization [56,57,58,59,60,61]. The vapour-phase polymerization of pyrrole is an additional method to form conducting PPy films on membranes [62,63,64,65]. Addition of surfactants such as sodium dodecylbenzensulphonate, sodium alkylnaphtalenesulphonate and sodium alkylsulphonate within the chemical oxidation reaction media gives higher PPy electro-polymerization efficiencies, larger electric conductivities, better fluxes and selectivity control in the composite membranes [60]. The resulting nanoporous membranes are able to tune their pore sizes by application of an electrical potential, whose strength is less than 1.1 V.

Tsai et al. [60] prepared a PPy-based nanoporous membrane with tuneable wettability from a polypyrrole film doped with dodecylbenzenesulfonate anions (DBS) and electropolymerized on a coated Si wafer. Due to the reorientation of DBS dopant molecules, the membrane surface wettability was tuned from a more hydrophobic behaviour (with a contact angle of 134°) to a less hydrophobic behaviour (with a contact angle of 107°) by application of low electrical potentials (from 0.7 to −1.0 V).

#### 2.2.3. Responsive Membranes Based on Carbon Nanotubes

The use of metal nanoparticles and carbon nanotubes (CNTs) as conducting elements is a valuable approach for the preparation of effective electro-sensitive materials to be used in several fields including drug delivery [66,67], liquid crystal displays [68], solar energy cells [69], conductive devices [70,71,72]. CNTs are commonly employed in hybrid polymer membranes to improve their performance in terms of fouling potential, permselectivity, and flux. The specific features of CNTs, such as well-defined structure, chemical bonding properties and high aspect ratio, concur to their interesting electro-mechanical properties that can improve the morphological, rheological, thermal, mechanical, and electrical properties of the host polymers. Commercially available and laboratory-scale produced single-walled (SWCNT), double-walled (DWCNT) and multi-walled (MWCNT) CNTs can be incorporated into final and/or intermediate polymer materials. The most important challenges in the preparation and effective utilization of CNTs in polymer membranes are an adequate interfacial adhesion between polymer matrix and CNTs and a homogeneous distribution of CNTs throughout the composite matrix in order to prevent their agglomeration [73]. Moreover, CNTs concentration limits and inhomogeneous orientation in membranes represent additional issues to be overcome. Some approaches to face these challenges include the use of surfactant molecules, polymer wrapping, long sonication times and chemical sidewall-functionalization in order to favour debundling and enhance hydrophilicity [74].

### 2.3. Electro-Conductive Membranes for Permeation Enhancement

**Figure 1:**
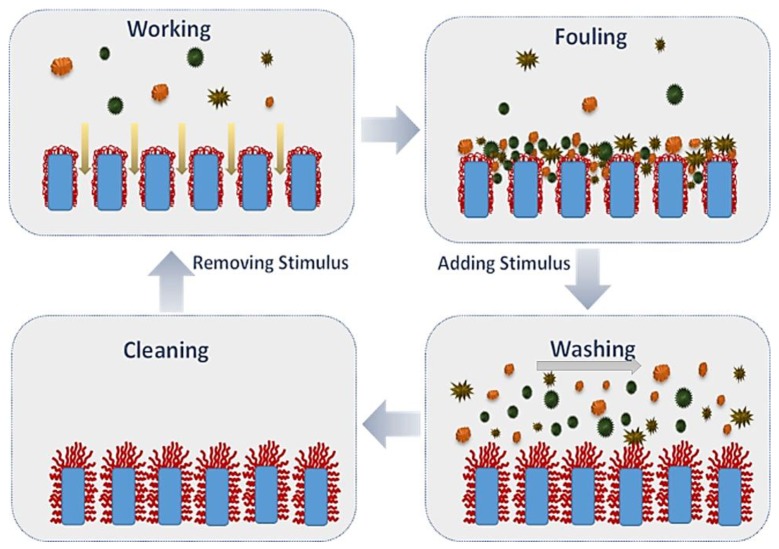
Examples of Stimuli Responsive Membranes. Reprinted from [83], with permission from Royal Society of Chemistry.

In recent years, the use of stimuli responsive membranes has become a promising method for reducing fouling potential. Treatments with stimuli responsive molecules in the form of thin films and nano-brushes give surface functionality to conventional membranes and reduce their fouling potential [75,76,77,78,79,80,81,82,83] (Figure 1). For example, it is possible to increase the membrane permeability and solve the problem of foulant deposition within pores by opening gates and enlarging pore size [84,85,86].

**Figure 2:**
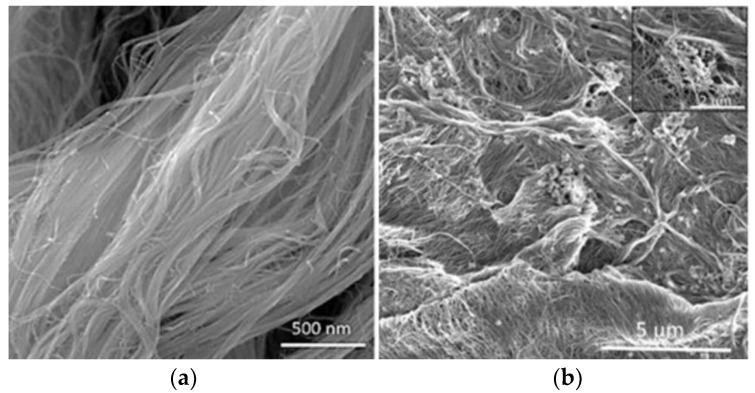
SEM images of carbon nanotube structures: (**a**) pure and (**b**) cast on PVDF membrane. Reprinted from [87], with permission from Elsevier.

Lalia et al. [87] proposed self-cleaning PVDF membranes by using highly tangled carbon nanostructures (CNS) with an average diameter of 7–8 nm. Membranes were characterized by improved processability, high electrical conductivity and large surface area [88] (Figure 2).

**Figure 3:**
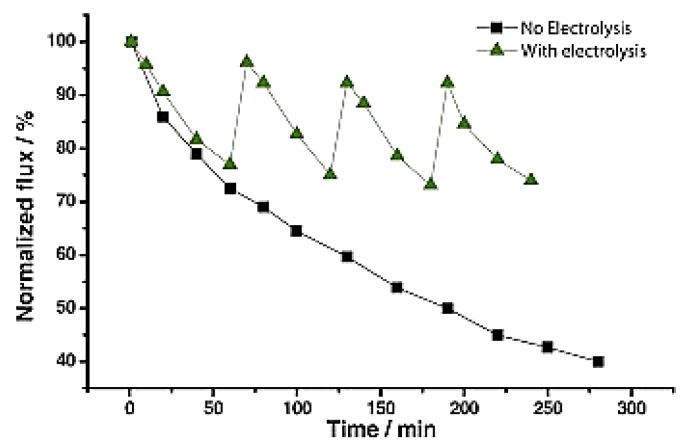
Time behaviour of normalized flux for CNS/PVDF membrane with and without electrolysis. Reprinted from [87], with permission from Elsevier.

These membranes were prepared via vacuum filtration, followed by a heat treatment at 160 °C to melt PVDF and provide binding sites inside the entangled CNS structure with the aim to improve the membrane mechanical strength. Then, membrane performance was tested for in situ surface cleaning in a cross-flow filtration using a yeast suspension as feed. In the electrolytic cleaning the CNS/PVDF surface acted as the cathode, a platinum foil was used as the anode and Ag/AgCl was employed as the reference electrode in 0.5 M H_2_SO_4_ solution. Electrolysis led to the generation of hydrogen micro-bubbles on the membrane surface, which removed foulants and recovered flux in successive cycles. Permeation fluxes exponentially decreased with time in absence of periodic electrolysis, while they increased of about 40% respect to their original values after 4.6 h of filtration in the presence of periodic electrolysis (Figure 3).

**Figure 4:**
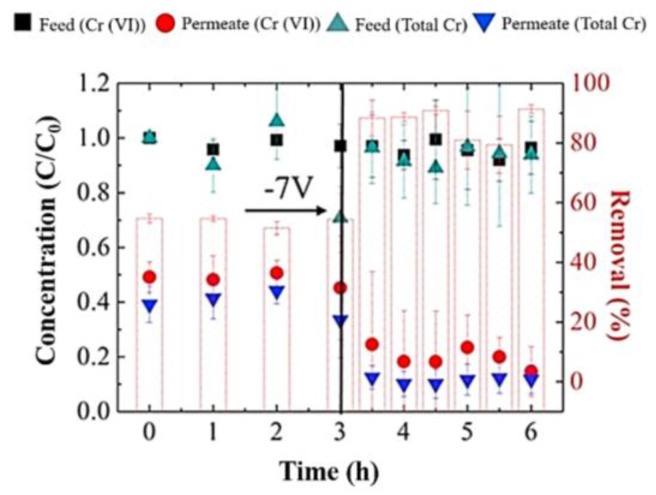
Removal of chromium from tap water spiked with 1 ppm Cr(VI) using a 6 µm-tick membrane. Reprinted from [89], with permission from Elsevier.

Recently, Duan et al. [89] used a polyvinyl alcohol and carboxylated MWCNTs (PVA/MWCNT-COOH) membrane to remove Cr(VI) from water through a combined process of electrostatic repulsion, electrochemical reduction, and precipitation. The overall removal efficiency exceeded 95%, a very high value if compared with the maximum rejection of 20% by commercial UF polysulfone membranes with a cut-off of 10 kDa. An electrochemical treatment of Cr(VI) is usually conducted in a mass-transport limited batch process that needs long contact times, making the process hard to scale up. These mass transfer restrictions can be overcome by electrochemical filtration, where the contaminated water is forced through a porous electrode, as a PVA/MWCNT-COOH membrane, capable of supporting electrochemical reactions, such as oxygen reduction, chlorine oxidation, and water splitting [90,91]. The removal mechanism resulted to be highly dependent on solution conductivity: higher solution conductivities involved electrochemical reduction and precipitation of Cr(III) on the membrane surface, while very low conductivities led to electrostatic repulsions accounting for Cr(VI) rejection from the permeate. The increase of membrane surface charge density by application of an external potential (3, 5 and 7 V, with membrane as cathode), increased the Cr(VI) removal from 45.0% (for non-polarized PVA/MWCNT-COOH membrane) to 86.5% (at the highest cell potential). The membrane contact time and background ionic strength of the feed solution influenced significantly the Cr(VI) removal. Electrostatic repulsive forces between the negatively charged PVA/MWCNT-COOH membrane and CrO_4_^2−^ could prevent chromium ions permeation under low salinity conditions without applying external potentials. At high electrolyte concentrations, soluble Cr(VI) is reduced to insoluble Cr(III) and precipitates on the membrane surface primarily as Cr(OH)_3_ by reaction with hydroxide ions generated by the water splitting on the MWCNT network, and can be removed from the treated water stream. Moreover, thicker membranes (6 μm-tick) showed superior performance with better rejection/removal and higher current densities, also when PVA/MWCNT-COOH membranes were used to treat tap water spiked with 1 ppm Cr (VI) by application of 7 V to the membrane counter electrode (Figure 4).

## 3. Membrane Fouling

Fouling can be considered the “Achilles heel” of membrane processes. It is essentially due to the deposition/adsorption of particulate and soluble materials on membrane surface and, in case of porous membranes, to the entrapment of foulant molecules inside membrane pores [92]. Several factors can influence fouling: the feed conditions (e.g., ionic strength, pH and presence of cations), membrane surface morphology and properties such as roughness, charge and hydrophilicity [93].

**Figure 5:**
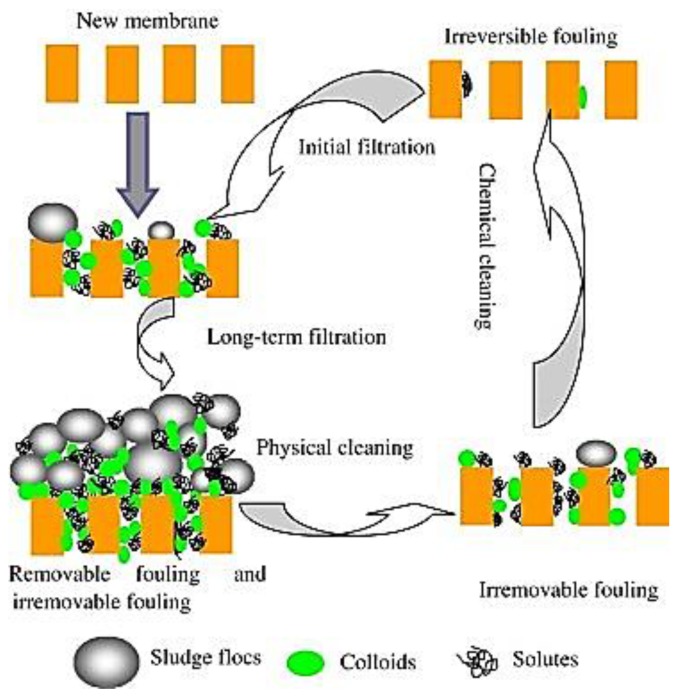
Reversible, irreversible and irrecoverable fouling in membrane processes. Reprinted from [92], with permission from Elsevier.

Membrane fouling can be essentially classified into three main categories: reversible, irreversible and irrecoverable, depending on the nature of foulant attachment onto membrane surface. Reversible fouling is caused by external deposition of material and can be removed by simple cleaning methods, while irreversible fouling refers to foulants, which can only be removed by harsh chemical and/or thermal treatments. The term irrecoverable fouling refers to fouling that cannot be removed by any cleaning method, but only by a long operational period [92] (Figure 5).

Another important classification divides fouling in abiotic fouling and biofouling. Abiotic fouling is responsible of the formation of a “cake layer” consisting of rejected material, while biofouling is the accumulation of microorganisms onto the surfaces and within the pores of membranes [94].

Fouling can significantly reduce membrane performance by:

1. a lowering in productivity because of longer filtration times,
2. an alteration of membrane selectivity as a consequence of a change in pore size,
3. a shortening of membrane life because of the severe chemical cleaning [95,96],
4. an increase of operational costs [97].

The material accumulated onto surface or within pores may reduce the membrane permeability and results in a general reduction of the permeate flux over time [94]. For constant pressure operations, where the transmembrane pressure is maintained at a constant value during filtration, fouling causes an increase in filtration resistance, that leads to a flux decline, *FD*, over time defined as [98]:

(2) $$FD=\;\frac{F_{i}-F_{f}}{F_{i}}\;\times \;100$$

where*F_i_* and *F_f_* are the initial and final fluxes, respectively.

The characteristics and the position of deposited materials determinate the extent and reversibility of permeate flux decline. A partial restore of permeate flux can be obtained by membrane cleaning (either by hydraulic or chemical methods) in order to remove some/all the accumulated material [94] (Figure 5).

**Figure 6:**
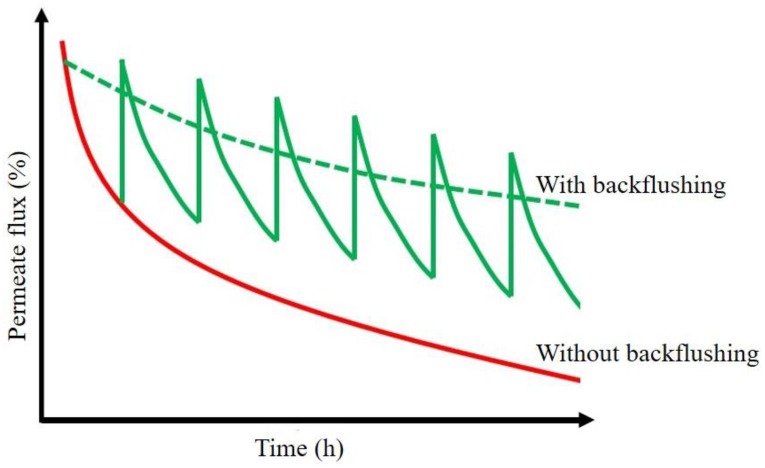
Effects of periodic back-flushings on permeate flux over time.

Conventional cleaning methods include back-flushing, feed pulsing, permeate back-pulsing and air sparging [99]. However, these methods have some limits because they can provide only a temporary relief to flux losses (Figure 6) and damage membranes [100] causing significant changes in their properties (e.g., surface charge, hydrophobicity and permeability) [101]. Further drawbacks are the increased operational costs, reduction of membrane lifetime, and need to interrupt the production to activate such cleaning procedures [99].

Obviously, a highly fouling-resistant membrane requires infrequent cleanings, reduces operating and disposal costs, increases the operational life and provides consistent permeate quality over time [94].

### 3.1. Novel Approaches to Mitigate Fouling

Extensive work has been done on developing methods to mitigate the negative effects of fouling on membrane performance including optimisation of the membrane composition to minimise attractive interactions between foulants and surface [102], pre-treatment to remove the most reactive foulants [103] and enhanced module design and operation that reduce fouling through a more effective hydrodynamics [104]. A novel approach suggested to prevent fouling is the formulation of membranes characterised by an active layer with a low surface energy so that attached foulants can be readily washed away with reduced changes in water fluxes and permeations [105].

Surface modification of commercial membranes by post-treatment is one of the most frequently method to decrease membrane fouling potential [106,107]. Some post-synthesis modifications include [94]:

1. a decrease in the membrane hydrophobicity,
2. a reduction of the surface roughness,
3. an increase in the membrane selectivity,
4. a modification of the surface charge.

Since main polymer membranes are hydrophobic, a frequent problem in membrane processes is the hydrophobic interactions between membrane surface and hydrophobic solutes present in the feed solution. The use of a hydrophilic membrane could decrease the fouling potential [92]. Numerous attempts have been made to improve anti-fouling performance by increasing membrane surface hydrophilicity and smoothness [6,108]. Du et al. [109] proposed a new post treatment process to increase membrane surface hydrophilicity and smoothness by a surface microstructure reassembly.

Recently, the attention of researchers has shifted to Electrically Responsive Polymer Membranes (ERPMs) characterised by high electrical conductivities. These self-cleaning membranes can be used to mitigate the effects of fouling in several types of separation processes [87]. The cleaning mechanism in ERPMs can be based on electrostatic repulsion, electrochemical oxidation, hydrogen peroxide production, surface morphology changes, piezoelectric vibrations, electro-chemical bubble generation [32,110,111,112,113].

#### 3.1.1. Electrostatic Repulsion and Hydrogen Peroxide Generation

A possible method to mitigate fouling consists in the generation of electrostatic repulsion between charged surfaces and foulants because most of the membrane foulants are negatively charged such as sludge flocs, soluble microbial products and polymer substances [114]. In addition, electrically charged membranes have been used as electro-catalytic platforms in order to transform various aqueous contaminants through electrochemical reactions [115].

**Figure 7:**
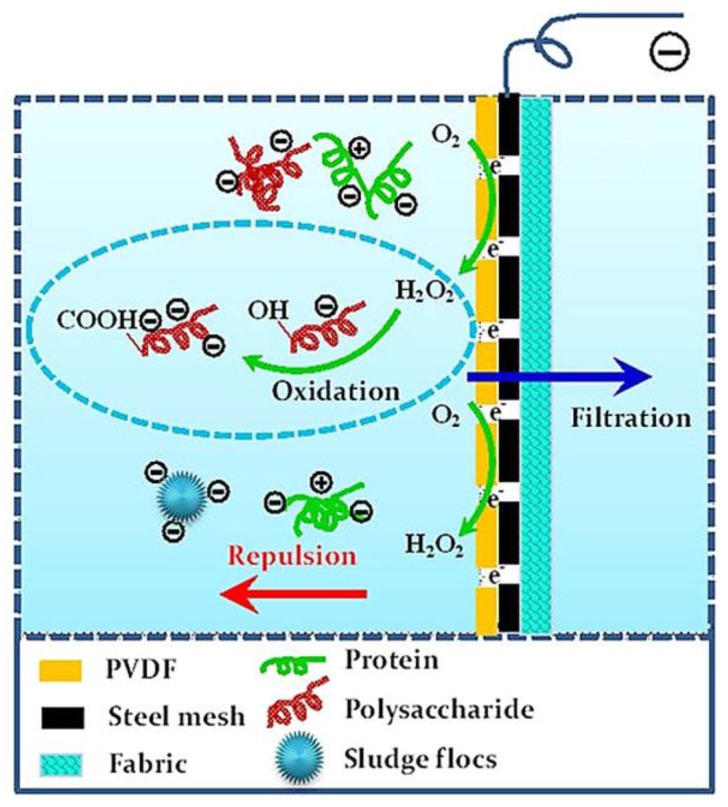
Schematic illustration of anti-fouling mechanism. Reprinted from [116], with permission from Nature Publishing Group.

Huang et al. [116] proposed a simple method to control fouling introducing a stainless steel mesh between the supporting layer and active layer of a MF polymer membrane without changing its surface physical-chemical properties. A homogeneous conducting polyvinylidene difluoride (PVDF) solution was cast on a stainless steel mesh (pore size 96 μm, thickness 43 μm) assembled on a polyester nonwoven fabric. The composite membrane was made by immersion precipitation in a non-solvent bath. Experiments were performed applying an electrical field of 2 V cm^−1^ with the membrane acting as cathode. A high water flux and low electrical resistance were found (66 L m^−2^ h^−1^ and around 200 Ω, respectively). The antifouling performance of these membranes was attributed to the combination of electrostatic repulsive forces between charged membranes and tested foulants, as well as to the organic oxidation by electrochemically generated hydrogen peroxide at the cathode (in situ membrane cleaning), leading to a decreased fouling potential (Figure 7). The electrical potential decreased the fouling rates for all tested model foulants (bovine serum albumin, sodium alginate, humic acid, and silicon dioxide).

CNTs are frequently used as additives in view of improving membrane performance [73,117]. In particular, electrically conductive membranes obtained by CNT entrapment in a crosslinked network have been demonstrated to mitigate several forms of fouling through the application of electrical potentials [118,119,120,121,122,123].

**Figure 8:**
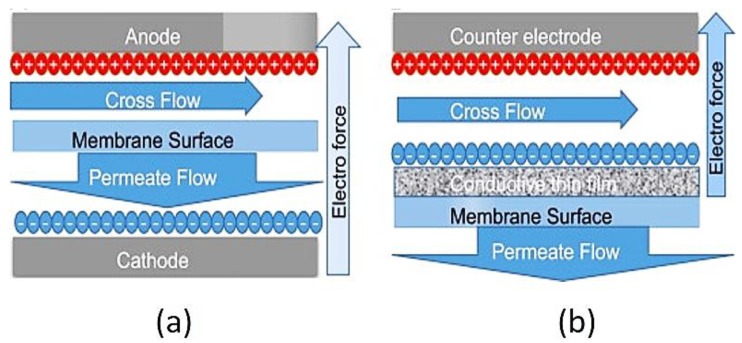
Electro-filtration set-up with: (**a**) conventional and (**b**) conductive membranes. Reprinted from [124], with permission from Elsevier.

Dudchenko et al. [124] used a sequential pressure/deposition process to set up robust and electrically conductive thin films made of glutaraldehyde-based cross-linked PVA and MWCNTs-COOH on a polysulfone UF support. This membrane exhibited high electrical conductivity (2500 S m^−1^), excellent robustness and permeability. PVA/MWCNT-COOH were used in cross-flow devices (Figure 8) for electro-filtration process and showed separation properties similar to the commercially available PSF-35 UF membranes.

When an electric potential was applied, PVA/MWCNT-COOH membranes were able to inhibit fouling at very high concentrations (3.0–5.0 g/L) of alginic acid, which was used as a negative charged model foulant. After 100 min of operation with the PVA/MWCNT-COOH membrane working as a cathode element (−3.4 V vs. Ag/AgCl reference electrode), the change in operating pressure was reduced by 51% compared with the control membrane working without voltage. Fouling mitigation was explained using a modified Poisson-Boltzmann equation and a DLVO-type theory, indicating that electrostatic interactions gave significant repulsive forces between the membrane surface and charged organic foulant molecules.

ERPMs have been demonstrated to be efficient in solving fouling problems in anaerobic bioreactors, when vigorous air scouring cannot be used to clean membrane surfaces [125,126].

**Figure 9:**
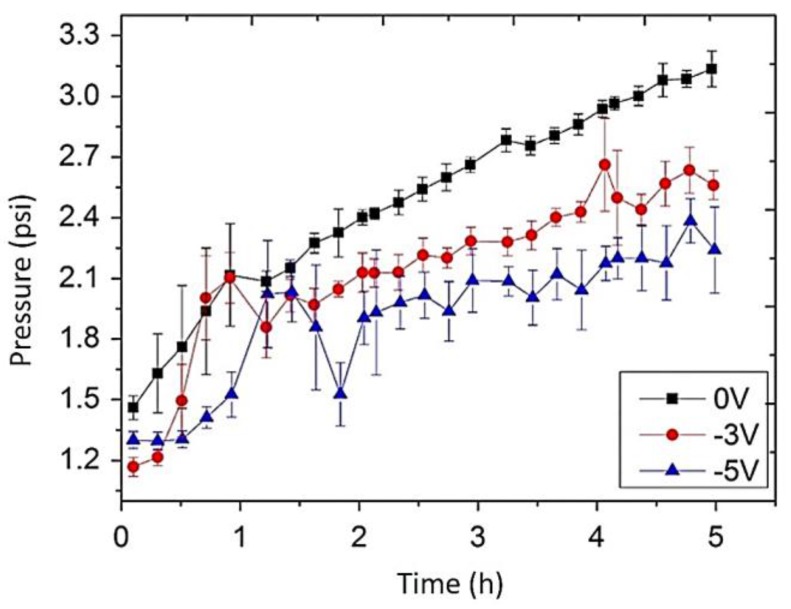
Impact of applied electrical potential on transmembrane pressure. Reprinted from [127], with permission from Elsevier.

Duan et al. [127] prepared a UF conductive membrane by deposition of CNT–COOH on a PSF support followed by the crosslinking of a PVA layer. The PVA/CNT–COOH network deposited on PSF surface created a smooth (46 ± 2 nm) electrically conducting (1132 ± 32 S m^−1^) layer. The application of an electric voltage (5 V) using membrane as cathode, led to a significant reduction of membrane fouling because the main degradation products of the anaerobic processes are negatively charged small molecules. When the system operated at a constant flux of 30 L m^−2^ h^−1^ with no applied potential, pressure increased from 1.5 to 3 psi over the time. On the contrary, when the membrane was used as a cathode, the pressure increased from 1.5 psi to 2.4 and 2.2 psi, when an electric potential of 3 V and 5 V was applied, respectively (Figure 9).

Interestingly, during back-flushing, when the membrane was anodically switched (1.5 V), a rapid and irreversible fouling was recorded confirming that most of foulants were negatively charged.

Another effective cleaning method to mitigate fouling without membrane damage is the generation of microbubbles on the membrane surface through electro-reduction [128].

#### 3.1.2. Electrochemical Oxidation

**Figure 10:**
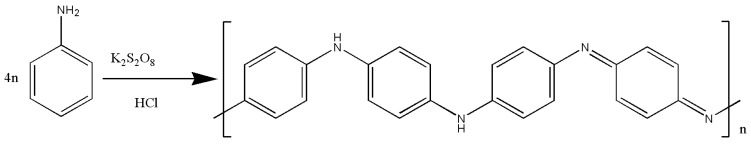
Emeraldine synthesis and structure.

The electrically conducting form of PANI is emeraldine, which is obtained through the electrochemical polymerization of PANI under acidic condition (Figure 10) [32,129,130,131,132].

PANI/CNT electrically conducting membranes were designed to evaluate their capacity for in situ electrochemical cleaning via electro-oxidation, without any external chemical addition [122].

**Figure 11:**
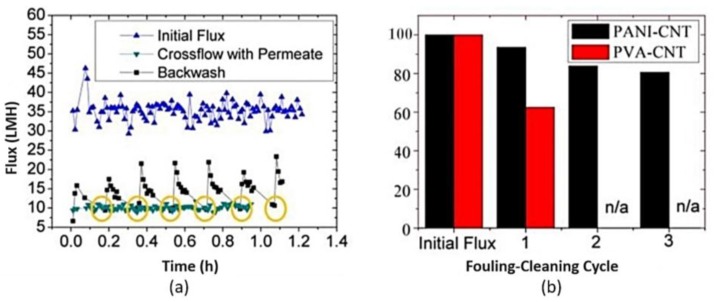
(**a**) Flux and (**b**) fouling behavior in PANI/CNT-COOH membranes. Reprinted from [122], with permission from American Chemical Society.

Recently, Duan et al. [122] made an highly conductive and anodically stable polyaniline/carboxylated carbon nanotubes (PANI/CNT–COOH) UF membrane by electro-polymerization of aniline on a PSF/CNT substrate under different acidic conditions (sulfuric, hydrochloric, and oxalic acid). Electrochemical polymerization under acidic conditions forms PANI in the emeraldine form. In addition, hydrophilic PANI-based membranes are usually more resistant to organic and biologic fouling as well as more conductive than PVA/CNT membranes. The PANI/CNT–COOH membranes obtained from sulfuric acid exhibited the best stability, conductivity and hydrophilicity with no impact on selectivity and permeability (Figure 11) and resulted ideal membranes for water treatment applications.

**Figure 12:**
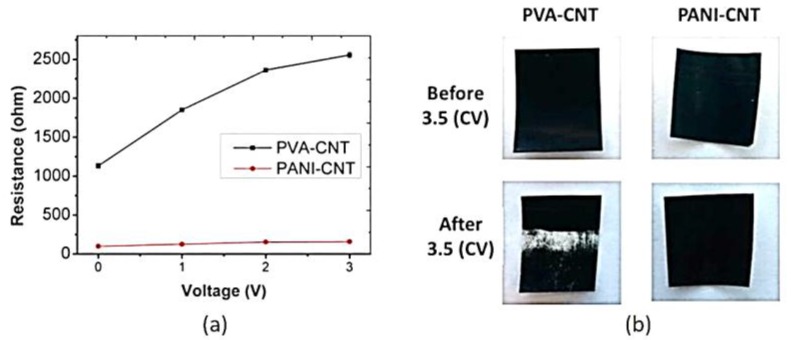
(**a**) Resistance and (**b**) stability in PANI/CNT–COOH and PVA/CNT-COOH membranes. Reprinted from [122], with permission from American Chemical Society.

Moreover, PANI/CNT–COOH membranes showed enhanced resistance to anodic oxidation, with little degradation observed up to 3 V vs. Ag/AgCl (Figure 12) under neutral pH conditions.

Experiments conducted with bovine serum albumin showed an easy fouling cleaning of PANI/CNT–COOH membrane surfaces by in situ oxidation and fluxes restored to their initial values by application of a 3 V potential. Moreover, a methylene blue (MB) solution was easily electrochemically oxidized with 90% efficiency in a single pass through an anodically charged ERPM (3 V, 1 μm thick membrane, membrane residence time lower than 0.2 s), avoiding the need for additional and expensive chemical cleaning agents [133]. The electro-oxidation of 5 ppm of MB on PANI/CNT–COOH ERPM required only 2.5 kW m^−3^ with a contact time lower than 1 s. In contrast, typical photocatalytic processes for MB on titanium dioxide require up to 40 kW m^−3^ and contact times ranging from 30 to 60 min [134,135].

Recently, many researches underlined the huge potential of CNT/polymer composites in water treatment such as desalination. Shawky et al. [73] synthesized polyamide/MWCNT nanocomposite membranes (PA/ MWCNT) by a polymer grafting process and investigated the NaCl and humic acid rejection. The strong interactions between MWCNTs and PA matrix resulted in a remarkable structural compactness and significant improvement of mechanical properties of the obtained membranes. In addition, the salt rejection considerably increased, even if the permeate flux was slightly reduced.

De Lannoy et al. [136] evaluated the effects of MWCNTs-COOH on a hydrophobic PSF membrane, widely used in UF processes in spite of its relatively low tensile strength. Surface hydrophilicity, membrane permeability and tensile strength of PSF/MWCNTs-COOH composites increased as a function of CNT carboxylation. However, a decreased MWCNT retention within the membranes and an increased leaching during membrane cleaning were observed at higher carboxylation efficiencies.

**Figure 13:**
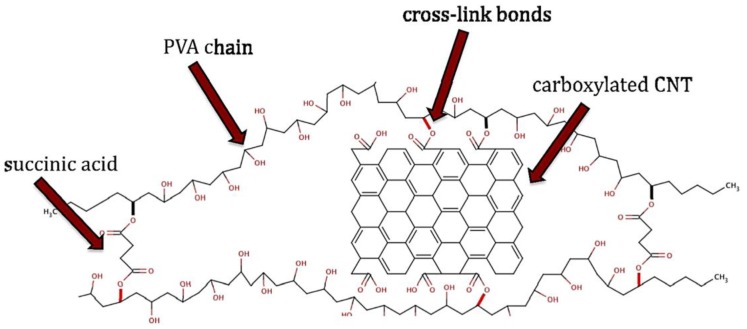
Thin layer of PVA covalently cross-linked to MWCNTs–COOH and succinic acid onto a cellulose nitrate membrane. The succinic acid molecules and MWCNTs–COOH cross-linked the PVA strands, immobilizing MWCNTs and altering the spacing between PVA strands. Reprinted from [137], with permission from Elsevier.

A highly conducting and flexible composite membrane was realized with a thin layer of PVA, covalently cross-linked to MWCNTs–COOH and succinic acid, onto a cellulose nitrate membrane (Figure 13) [137].

**Figure 14:**
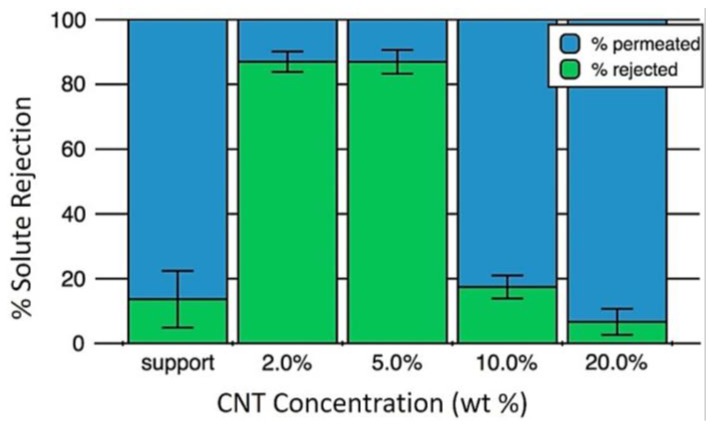
Solute rejection as a function of MWCNT–COOH wt % content with respect to PVA in PVA/MWCNT–COOH composites. Reprinted from [137], with permission from Elsevier.

This PVA/MWCNT–COOH composite showed high electrical conductivity and permeate flux with low polymer crystallinity and surface tension. Membranes prepared with 20 wt % MWCNT–COOH and 20 min curing time exhibited conductivities as high as 3.6 × 10^3^ S m^−1^, pure water flux of 1440 L/m^2^ h at pressures of 550 kPa, and triple-point initial contact angles as low as 40° with high hysteresis. Better separation characteristics were achieved in PVA/MWCNT–COOH membranes by incorporating smaller amounts of MWCNT–COOH (2 and 5 wt %), but at the expense of the membrane permeability (Figure 14). The authors suggest that the MWCNT-COOH loading could be easily employed to control the molecular weight cut-off.

CNT-based ERPMs show long-term stability (no notable change in their conductivity over time is observed when they are used as cathodes) and interesting electro-cleaning properties, as previously reported. Nevertheless, CNT-based ERPMs result to be unstable under elevated anodic potentials in aqueous environments due to CNT oxidation and breakdown when exposed to hydroxyl radicals produced on their surfaces [122]. Coating and anchoring of stable metal nanoparticles (e.g., bismuth-doped tin oxide and cobalt oxide) on CNT surfaces increase stability up to 2.2 V vs. Ag/AgCl reference electrode.

Graphene is a two-dimensional, one-atom-thick layer of graphite with tunable size and structure and can be engineered for different filtration processes, ranging from ultrafiltration to reverse osmosis. Graphene shows enhanced physical-chemical properties such as electrical and thermal conductivity, mechanical strength, optical transparency, solution processability, and specific surface area up to 2630 m^2^ g^−1^. Therefore, graphene has been widely used in flexible transparent electrodes, energy storage devices, solar cells, and electronics and optoelectronics applications. Usually, graphene can be obtained by exfoliation of highly pure graphite and, therefore, does not retain the CNTs metal impurities deriving from their metal-catalysis-driven growth process. Graphene nano-platelets (GNP) can lead to highly ordered membranes or films by means of different routes (filtration-assisted assembly, chemical vapor deposition, electrochemical deposition, and layer-by-layer methods). Moreover, the rich surface chemistry of bidimensional graphene favors the fine-tuning of the interfacing properties with numerous porous supporting materials, such as PSF, PES (polyethersulfone) and PTFE (polytetrafluoroethylene).

**Figure 15:**
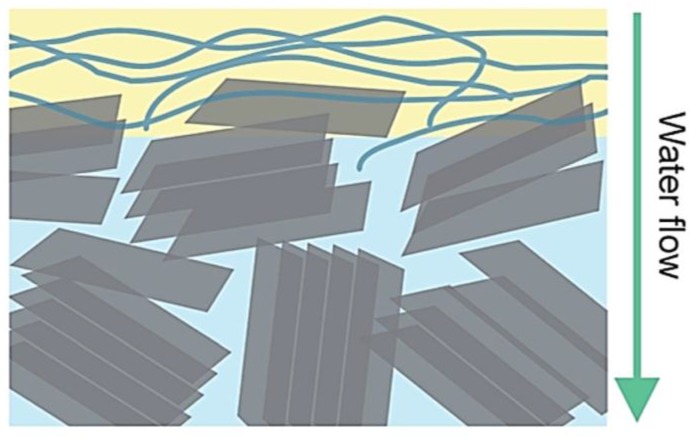
Schematic representation of PTFE/GNP:CNT electrochemical filter. Reprinted from [138], with permission from Royal Society of Chemistry.

Liu et al. [138] developed a novel electrochemical filter for water purification by graphene nano-platelets enabled by carbon nanotubes (GNP:CNT) in a polytetrafluoroethylene membrane (PTFE/GNP:CNT). CNTs were the conductive binders for graphene nano-platelets (Figure 15).

In particular, the researchers dispersed different weight ratios (from 50:50 to 100:0) of GNPs and CNTs in *N*-methyl-2-pyrrolidone, and vacuum filtered the stable suspension onto a PTFE membrane. Anodic oxidation of the PTFE/GNP:CNT electrode was tested using ferrocyanide ($\mathrm{Fe}{\left(\mathrm{CN}\right)}_{6}^{-4}$) as a model electron donor. When the anodic filter was used in batch mode, electro-oxidation rates increased linearly with applied potential and plateaued because of mass transfer limitations. When the PTFE/GNP:CNT filter was evaluated as part of a flow-through system, no plateau was observed for high concentrations of ($\mathrm{Fe}{\left(\mathrm{CN}\right)}_{6}^{-4}$) (10 mmol L^−1^) as a result of increased mass transfer rates. Overall, electro-oxidation rates increased up to 15-fold due to convection enhanced transfer of the target molecule to the electrode surface and reduction of mass transfer over potential.

**Figure 16:**
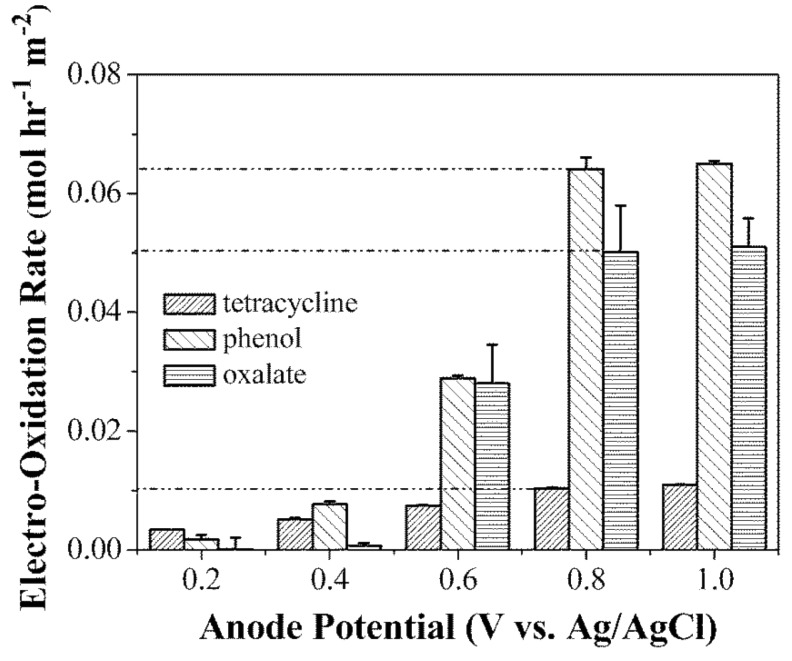
Electro-oxidative filtration of tetracycline, phenol and oxalate as a function of anode potential. Reprinted from [138], with permission from Royal Society of Chemistry.

Moreover, the efficiency of PTFE/GNP:CNT filters for anodic degradation was evaluated with three selected organic pollutants (tetracycline, phenol and oxalate). For all three organic compounds, electro-oxidation kinetics increased with increasing anode potential until a maximum removal rate (0.010, 0.064, and 0.050 mol h^−1^ m^−2^ for tetracycline, phenol, and oxalate, respectively) achieved at 0.8 V (Figure 16).

### 3.2. Biofouling Mitigation with Electro-Responsive Membranes

Membrane processes are vulnerable to bacterial adhesion and biofilm growth on the membrane surface. Development of biofouling leads to a dramatic decrease of productivity, especially when in the feed solution are present organic matter and nutrients, as in the case of wastewater effluents. Most efforts to prevent biofilm development are based on limiting the initial bacterial attachment or increasing detachment. Methods to develop surfaces with anti-biofouling properties include linking and embedding of antimicrobial nanoparticles [139,140,141,142], grafting of polymer brushes, that form a hydrated gel layer that prevents bacteria from interacting with surfaces [143], and electrically charged surfaces [144,145]. These coating methods have been demonstrated to be effective, but the coating material loss causes the decline of their performance over time [146,147,148]. Ronen et al. [120] studied the bacterial deposition and detachment rates as a function of the electrical potential applied to the membrane surface. In the experiment, the authors used a conducting PVA/CNT composite UF membrane and ITO electrodes positioned on both sides of a modified cuvette containing *Escherichia Coli* suspension at a concentration of 10^8^ cells mL^−1^. The microbial attachment was investigated using a direct observation cross-flow membrane system mounted on a fluorescent microscope. Different electrical potentials (0, 0.5, 1, and 1.5 V) were applied to the electrodes and the impact of the electric voltage was investigated by measuring cell integrity and cell viability using propidium iodide and 5-cyano-2,3-ditolyltetrazolium chloride as fluorescent indicators, respectively.

**Figure 17:**
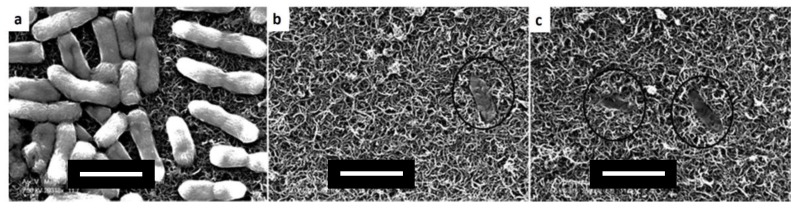
SEM images of membranes after detachment with: (**a**) no applied potential, (**b**) 1.5 V applied with membrane as anode, and (**c**) 1.5 V applied with membrane as cathode. Scale bars are 2 µm. Reprinted from [120], with permission from American Chemical Society.

SEM images of membrane surface after detachment phase showed that cells had a regular shape on membranes without applied potential, while the irregular structure of cells, that were remained attached to membrane subjected to a potential of ±1.5 V, was indicative of cell damage (Figure 17).

The main mechanism proposed to explain the antifouling properties of these membranes was the generation of hydrogen peroxide due to electro-reduction of oxygen, when a low electrical potential was applied. The production of hydrogen peroxide on membrane surfaces caused the reduction of microbial cell viability, increased cellular permeability and prevented bacterial attachment.

Another interesting anti-biofouling method based on the electrostatic repulsion between membrane surface and attached bacteria was investigated by Baek et al. [149]. They produced an electro-conductive feed spacer (ECFS) in a lab-scale cross flow membrane system, in which low electric potentials were applied to minimize chlorine gas generation. A titanium mesh and a stainless steel mesh were used as model ECFS, on which an electrical polarization was induced. After 24 h from biofouling occurrence, the permeate flux was decreased to about 47%, while was recovered to 80%, 89%, and 91% when the ECFS was polarized for 30 min with +1.0 V, −1.0 V, and alternating electrical potentials (cycles of +1.0 V for 1 min and −1.0 V for 1 min), respectively.

The electrically conductive PA/MWCNT nanocomposites by de Lannoy et al. [74] showed higher biofilm-preventing capabilities, larger electrical conductivity (∼400 S m^−1^), better monovalent ion rejection (greater than 95%), and higher water permeability than commercially available membranes. Biofilm induced a non-reversible flux decline, while the flux decrease for membranes with an applied electric potential (1.5 V) was lower (due just to bacteria deposition) and fully recoverable with a short rinse with the feed solution without added cleaning agents. The prevention of microbial biofilms was probably due to local pH instabilities and unfavourable conditions for bacterial growth arising from the electrical potential.

## 4. Conclusions

This paper reviewed the recent progresses in electro-conductive membranes as smart devices able to respond to the application of an electric signal. Different classes of electro-conductive membranes were examined and the advantages in their using were discussed with particular emphasis to the beneficial effects on membrane transport properties and fouling mitigation.

## Figures and Tables

**Table 1:** Some electro-conductive polymers and their abbreviations.

Chemical Name	Abbreviation
Polyacetylene	PAc
Polyaniline	PANI
Polyazulene	PAZ
Polybutadiene	PBD
Polyisopren	PIP
Poly(isothianaphtene)	PITN
Polyfuran	PFu
Poly(α-naphthylamine)	PNA
Poly(p-phenylene)	PPP
Polythiophene	PTh
